# Delivering stepped care: an analysis of implementation in routine practice

**DOI:** 10.1186/1748-5908-7-3

**Published:** 2012-01-16

**Authors:** David A Richards, Peter Bower, Christina Pagel, Alice Weaver, Martin Utley, John Cape, Steve Pilling, Karina Lovell, Simon Gilbody, Judy Leibowitz, Lilian Owens, Roger Paxton, Sue Hennessy, Angela Simpson, Steve Gallivan, David Tomson, Christos Vasilakis

**Affiliations:** 1 Mood Disorders Centre, Washington Singer Building, University of Exeter, Exeter, EX4 4QG, UK; 2 Health Sciences Research Group, Manchester Academic Health Science Centre, University of Manchester, M13 9PL, UK; 3 Clinical Operational Research Unit, University College London, Gower Street, London WC1E 6BT, UK; 4 Camden and Islington NHS Foundation Trust, St Pancras Hospital, 4 St Pancras Way, London, NW1 0PE, UK; 5 Centre for Outcomes Research and Effectiveness, University College London, 3rd floor, 1-19 Torrington Place, London, WC1E 7HB, UK; 6 School of Nursing, Midwifery and Social Work, University of Manchester, University Place, Jean McFarlane Building, Oxford Road, Manchester, M13 9PL, UK; 7 Department of Health Sciences, University of York, University Road, Heslington, YORK, YO10 5DD, UK; 8 'No Panic', 93 Brands Farm Way, Telford, Shropshire, TF3 2JQ, UK; 9 Newcastle, North Tyneside and Northumberland Mental Health Trust, Modular Building, St Nicholas Hospital, Jubilee Road, Newcastle upon Tyne, NE3 3XT, UK; 10 Collingwood Surgery North Shields, Hawkeys Lane, North Shields, NE65XH, UK

## Abstract

**Background:**

In the United Kingdom, clinical guidelines recommend that services for depression and anxiety should be structured around a stepped care model, where patients receive treatment at different 'steps,' with the intensity of treatment (*i.e*., the amount and type) increasing at each step if they fail to benefit at previous steps. There are very limited data available on the implementation of this model, particularly on the intensity of psychological treatment at each step. Our objective was to describe patient pathways through stepped care services and the impact of this on patient flow and management.

**Methods:**

We recorded service design features of four National Health Service sites implementing stepped care (*e.g*., the types of treatments available and their links with other treatments), together with the actual treatments received by individual patients and their transitions between different treatment steps. We computed the proportions of patients accessing, receiving, and transiting between the various steps and mapped these proportions visually to illustrate patient movement.

**Results:**

We collected throughput data on 7,698 patients referred. Patient pathways were highly complex and very variable within and between sites. The ratio of low (*e.g*., self-help) to high-intensity (*e.g*., cognitive behaviour therapy) treatments delivered varied between sites from 22:1, through 2.1:1, 1.4:1 to 0.5:1. The numbers of patients allocated directly to high-intensity treatment varied from 3% to 45%. Rates of stepping up from low-intensity treatment to high-intensity treatment were less than 10%.

**Conclusions:**

When services attempt to implement the recommendation for stepped care in the National Institute for Health and Clinical Excellence guidelines, there were significant differences in implementation and consequent high levels of variation in patient pathways. Evaluations driven by the principles of implementation science (such as targeted planning, defined implementation strategies, and clear activity specification around service organisation) are required to improve evidence on the most effective, efficient, and acceptable stepped care systems.

## Background

Evidence-based medicine (EBM) is the 'conscientious, explicit, and judicious use of current best evidence in making decisions about the care of individual patients' [1]. Production of clinical guidelines is a conventional method of operationalising EBM and ensuring that clinical and cost-effective 'health technologies' are used in routine service settings. However determining the clinical and cost-effectiveness of health technologies does not provide a blueprint for their delivery in practice [2]. Service delivery and organisation is the focus of health services research (HSR), which aims to "identify the most effective ways to organize, manage, finance, and deliver high-quality care; reduce medical errors; and improve patient safety" http://archive.ahrq.gov/about/whatis.htm.

In the United Kingdom (UK), the National Institute for Health and Clinical Excellence (NICE) clinical guidelines for depression [3,4] used evidence synthesis to summarise available data on the effectiveness of individual health technologies and outline 'what works for whom.' However, despite their basis in health technology assessment, the guidelines also made strong recommendations about service delivery and organisation, suggesting that services for depression should be structured around a stepped care model [5,6].

**Figure 1:**
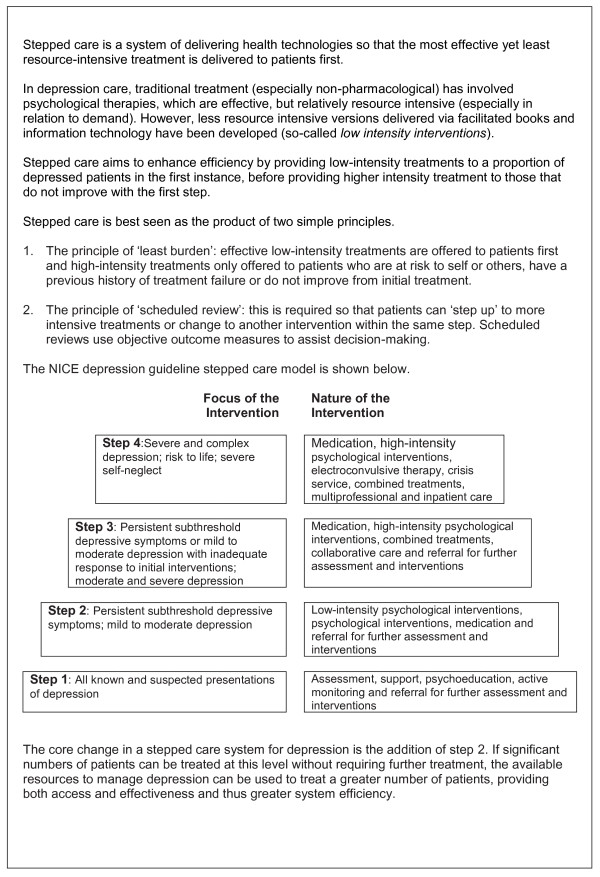
**Stepped Care**.

One class of NICE recommended treatment--psychological therapy--is acceptable and effective for depression, but access to it is often problematic because of limited numbers of trained professionals to deliver this treatment. Stepped care is a model that seeks to ameliorate problems with access through better allocation of scarce psychological therapy resources. This is achieved through use of 'low-intensity' psychological interventions that deliver psychological help through written self-help books or computer platforms (*e.g*., http://www.moodgym.anu.edu.au), supported by limited professional contact. These require less input from a trained therapist [7] than conventional 'high-intensity' face-to-face treatments, such as brief psychological therapies (involving 6 to 12 sessions with a therapist), and even more intensive long-term therapies (involving 16 or more sessions--see Figure 1).

The adoption of the stepped care model within all NICE guidelines for common mental health problems, including now anxiety [8], is a reasoned response to the challenges of effective depression care, but was not based on the same, detailed evidence synthesis as recommendations about individual treatments. There are very limited data available on the operation of the stepped care model and significant questions remain about implementation [9]. These include the optimal number of steps and the range of treatments within steps; the proportion of patients who might bypass low-intensity treatments and be referred directly to higher intensity treatments; the process of decision-making about 'stepping up' to higher intensity of care; and the degree to which stepped care systems should be responsive to local context.

One of the core tensions for those designing services for depression is the balance between 'stepped' and 'stratified' models of care. Each model is based on the idea that depression services should deliver both low-intensity and high-intensity treatments. However, the way in which patients access those different interventions is more contested. In a stepped model, the system is self-correcting, [5,6] in that although most patients are assigned to low-intensity interventions initially, those failing to benefit are 'stepped up' to higher intensity treatment. This has the advantage of maximising the proportion of patients who might benefit from low-intensity interventions, but may potentially delay access to appropriate care for some patients. In contrast, a stratified model seeks to assign patients to particular steps on the basis of their presenting characteristics (such as initial depression severity) to better target interventions at patients likely to benefit [10]. This is potentially a better method to ensure timely delivery of the appropriate intensity of intervention, but is dependent on accurate knowledge of the types of patients who are most likely to benefit from a particular intensity of intervention (so-called 'aptitude treatment interactions') [6]. Although a combination of 'stepping' and 'stratification' is likely to be required, the relative importance of the two different mechanisms was not explicit in the NICE guidelines.

The NICE guidelines have underpinned a six-year £700 m UK investment in psychological therapies, the Improving Access to Psychological Therapies (IAPT) programme http://www.iapt.nhs.uk. Understanding implementation is thus a priority to ensure effective use of these resources. We wanted to investigate how mental health services in the UK implemented the service delivery and organisation recommendations of NICE so that we could provide advice to policy makers on the likely shape of stepped care service delivery systems in routine practice.

### Aims

We aimed to describe the operation of stepped care in a number of sites in the UK in order to answer the following questions:

1. What different models of stepped care are implemented in practice?

2. How do patients access and flow through the different models?

3. What proportion of patients are managed at each step, and what proportion 'stepped up'?

## Methods

The study was part of a larger operational research project to develop a decision and modelling aid for services designing stepped care organisational systems http://www.sdo.nihr.ac.uk/files/project/109-final-report.pdf.

The study was observational in design, using routine data in four NHS service sites consisting of between 22 and 46 mental health workers. Each site was recruited to this study on the basis that they were aiming to set in place services to provide patients with a low- and high-intensity treatment options as described in the NICE guidelines, organised in a stepped care manner within the context of their existing resources and personnel. In the UK, during the data collection period, mental health services for people with depression and anxiety were not delivered to a standard blueprint but could be provided by a range of National Health Service (NHS) organizations including primary care or specialist care NHS Trusts. In some cases all treatment services could be provided by one organization that may be a primary or specialist care organization. In other cases, several organizations could provide different elements of a psychological therapies service.

In a previous stage of the project (see http://www.sdo.nihr.ac.uk/files/project/109-final-report.pdf), we facilitated a consensus workshop at each site to help teams design their stepped care models. We used the 'constituency approach' [11] to enable sites to build consensus gradually, providing participants with skilled facilitation around clear inputs and defined outputs in a carefully structured process [12]. Our team provided the structured environment for sites to reach their own design consensus, at no point influencing the sites' specific choices of new service configuration. Following this exercise, sites set up implementation teams locally to restructure their services.

### Data collection

Our conceptual framework was the 'patient pathway' [13], *i.e*., the route that a patient will take from their first contact with a clinical service, including referral, to the completion of their treatment. Given the potential diversity of organizations able to deliver these services in the NHS, we recorded service level descriptors for each site, including which professionals referred into the stepped care system; who made the initial assessment of the patient; how patients were allocated to steps; whether self referral was permitted; and whether there was a single point of entry to the system for all patients, or whether patients could 'bypass' aspects of the service. Where possible we extracted individual level patient data from information systems used by sites to manage their activity, although clinicians were asked to collect additional information on treatments received by patients where existing data systems did not collect this. All sites used different information systems and had varying levels of information collected by clinicians. We collected data on how and when patients in each site accessed, received and transited between the various treatment options, including the type of treatments, the step/intensity of the treatments, and the patient's final status at the end of the project time (*i.e*., dropped out of treatment, completed treatment or still remained in treatment). These data were extracted from information systems and supplementary record sheets onto a standard proforma by clerks in each site, trained and supervised by project research workers who also validated their data extractions against source data. We also recorded demographic data on patient gender, age, ethnicity, employment status, main mental health problem identified by clinicians, and medication use.

### Analysis

Data were anonymised by local NHS service data clerks and submitted to the research team as a flat database file and cleaned. We analysed categorical data, including demographics as frequency counts and percentages (with ranges where applicable), continuous data as means, and standard deviations. For those variables where there were missing data, we excluded these cases from the analysis of that variable, and the total number of cases used for each variable is presented in the results with percentages of data missing. We analysed information about patient pathways using start and end points and whether they were stepped up or down. We then mapped patient flow visually to illustrate patient movement through these exemplar stepped care systems.

The limited time span of the project did not allow us to collect final endpoint status data about the proportion of patients who remained in treatment at the end of the project, particularly in high-intensity treatment which by its nature is of longer duration than low-intensity.

### Ethical approval

Ethical approval for the study was given by the South West Multisite Ethics Committee in the UK (05/MRE06/71).

## Results

### Setting

Table 1 describes population characteristics in each site. Some sites were part of a specialist mental health organisation while others were managed by primary care services. In sites A, B, and C, there were between 22 and 25 staff conducting assessments and treatments. In site D there were 46 workers involved. Across all four sites, workers included para-professional mental health workers (*i.e*., mental health workers without a formal professional qualification), and professionally qualified workers including nurses, social workers, occupational therapists, and clinical psychologists. Professionally qualified workers delivered treatments at all steps, whereas para-professional workers delivered treatment at lower steps (Table 2).

**Table 1:** Population characteristics of the sites

	Type of organisation	Average IMD Rank^a^	Population
**Site A**	Specialist Trust		1.4 million
**Northern Urban/Rural**	Primary care Trust A	79	
	Primary care Trust B	32	
	Primary care Trust C	91	

**Site B**	Primary care Trust	78	750,000
**Northern Urban**			

**Site C**	Specialist Trust		570,000
**Southern Urban/Rural**	Primary care Trust D	149	
	Primary care Trust E	126	

**Site D**	Specialist Trust		500,000
**Southern Urban**	Primary care Trust F	35	
	Primary care Trust G	7	

^a^The Index of Multiple Deprivation (IMD) 2007 combines a number of indicators, chosen to cover a range of economic, social and housing issues, into a single deprivation score for each small area in England. This allows each area to be ranked relative to one another according to their level of deprivation. 1 indicates the most deprived area. IMD rank is shown out of a total of 152 Primary Care Trusts (PCTs).

**Table 2:** Characteristics of stepped care services by site

	Site A Northern Urban/Rural	Site B Northern Urban	Site C Southern Urban/Rural	Site D Southern Urban
**Site**	Specialist Trust-led	Primary Care-led	Specialist Trust	Primary Care led with Specialist Trust partner

**Assessment**	Qualified mental health practitioner face to face	Para-professional or qualified mental health worker face to face	Senior mental health worker in a GP based clinic triaging direct to steps from written information or following telephone or face to face appointment	Para-professional or psychologist face to face

**Step 2 low-intensity**	Guided self-help delivered by para-professionals; group classes by para-professionals and qualified mental health workers	Guided self-help delivered by para-professionals; group classes by para-professionals and qualified mental health workers	Self-directed cCBT,^1 ^guided self-help and group classes by para-professionals and qualified mental health workers	Guided self-help delivered by para-professionals, cCBT, group classes by para-professionals

**Step 3 high-intensity**	Short-term evidence-based psychological interventions delivered by a mental health practitioner or practice-based counsellor	Short-term psychological interventions including brief CBT delivered by a mental health practitioner	Short-term psychological interventions including brief CBT and group work delivered by trained para-professionals and mental health practitioners	Psychology, and counselling

**Step 4 high-intensity**	Complex evidence-based psychological interventions delivered by psychological services, CMHT, ^2 ^or the psychiatric service	Specialised psychological treatment delivered by community mental health teams, psychology and psychotherapy services working within the specialist mental health trust	Not specified	Psychology, psychotherapy and community mental health teams

**Step 5 high-intensity**	Crisis teams, self-harm liaison and in-patient admission by specialist clinical teams	Crisis teams, self-harm liaison and in-patient admission by specialist clinical teams	Not specified	Not specified

^1 ^cCBT: computerised Cognitive Behaviour Therapy^; 2 ^CMHT: Community Mental Health Team

### Models of stepped care

Each of the sites had developed a model of stepped care within their available resources that they regarded as reflecting their local contexts and best serving their local population needs (Table 2). All sites allowed patient self-referral, but most restricted this to access to low-intensity treatment steps (*i.e*., patients could self refer for access to written self help materials and computer based treatments at step two, but not treatments involving access to contact with mental health workers at steps three, four, and five). All sites allowed a range of professionals--general practitioners (GPs), other primary care staff, and secondary mental healthcare staff--to refer, although site A also accepted referrals from the UK 'third sector' (*i.e*., non-governmental organisations who deliver mental health services). The majority of patients were assessed by para-professional workers in the sites, although site C offered a triage service run by professionally qualified mental health workers (*e.g*., mental health nurses) who made decisions about allocation to all steps including a computerised treatment programme that was designed to be used by the patient independent of any contact with the service--'step one.' Sites A, C, and D allowed professionals to refer to different steps based on their clinical assessment; site B had a single point of entry (with a few exceptions) with the intention that all patients would be initially allocated to low-intensity treatments.

### Demographic characteristics of the patients

We collected data on almost 7,698 patients referred to four NHS sites operating stepped care services. Large amounts of demographic data were missing from service information systems at all sites, including age (75% missing), gender (60% missing), ethnicity (70% missing), employment status (42% missing), previous history (55% missing), identified problem (42% missing), medication status (54% missing), and sickness status (47% missing). On the basis of the available data, the patient populations accessing the stepped care services were largely female (63% to 67% across sites) and aged 20 to 39 (56% to 62%) with most employed (50% to 62%). Ethnic minority rates varied more widely (81% to 96% caucasian British). Absence from work through illness also varied widely (11% to 27%), with between 41% and 66% reporting a previous treatment history for anxiety or depression and between 48% and 65% taking psychotropic medication. All patients with available data were identified as experiencing anxiety, depression, or a mixed combination.

### Service delivery and patient pathways

**Figure 2:**
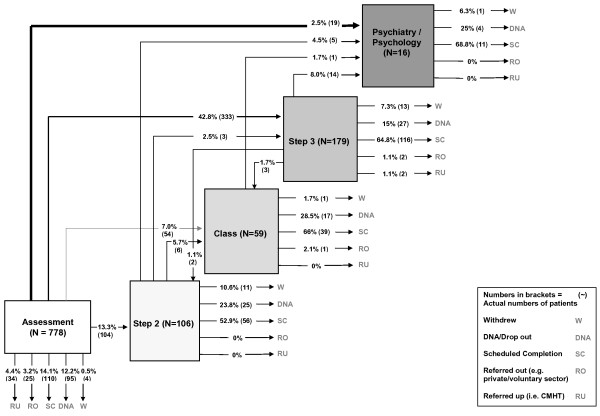
**Site A Patient Pathways**.

**Figure 3:**
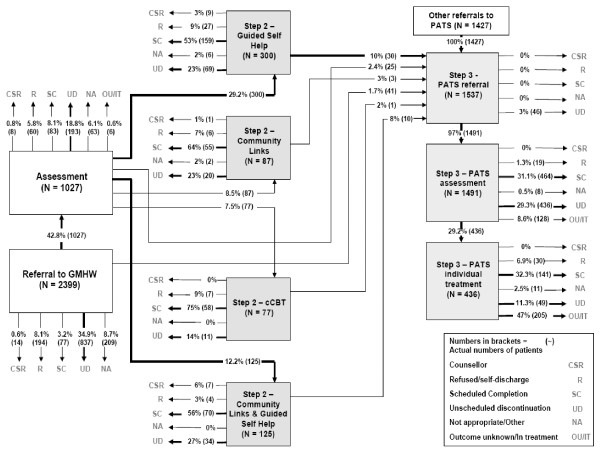
**Site D Patient Pathways**.

We were able to obtain complete information on patient flow and final status for all 7,698 patients. Sites had a wide range of referral numbers ranging from approximately 1,000 to nearly 4,000 in just over 12 months, with a variety of different patient flow results seen in each site (Table 3). Patient pathways were highly variable and complex both between and within sites (see Figures 2 and 3 for example maps of flows in sites A and D; more at http://www.sdo.nihr.ac.uk/files/project/109-final-report.pdf) with patients following multiple routes with multiple inputs and outcomes. For example, some patients entered and exited services at the same step; others entered the same step through different referral and assessment routes or made multiple transitions between steps. Some patients were assessed and treated entirely at high-intensity steps; others went through a low-intensity step first.

**Table 3:** Summary of number of patients referred and accessing each step by site

Activity	Site A	%	Site B	%	Site C	%	Site D	%
**Referral**	1,043		1,644		Not recorded		3,826	

**Assessment^a^**	778	75%	1,291	79%	1,185	N/A	2,518	66%

**Step 1^b^**	N/A	N/A	N/A	N/A	607	51%	N/A	N/A

**Step 2^b^**	162	22%	776	60%	178	15%	589	23%

**Step 3^b^**	336	43%	298	23%	40	3%	436	17%

**Step 4^b^**	39	5%	75	6%	N/A		N/A	

^a^as percentages of patients referred

^b^as percentages of patients assessed

NB: percentages do not add to 100 as patients may access more than 1 step.

**Figure 4:**
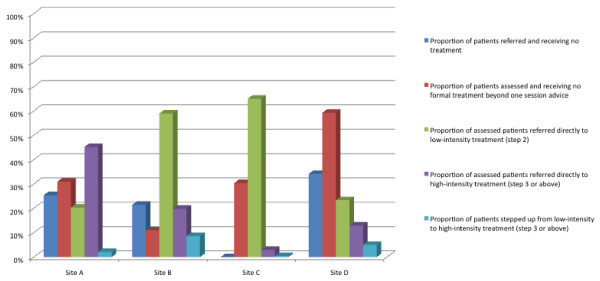
**Summary of Patient Pathway Data between Sites**.

Figure 4 summarises proportions of patients accessing different steps by different routes across sites. In some sites, allocation and direct referral to high-intensity steps were implemented, in line with a stratified model. This is most clearly illustrated in site A, where 45% of assessed patients directly accessed step three and received conventional high-intensity psychological therapy. However, adoption of this approach does not guarantee actual access to such treatment. In site C, after allocation, a lack of high-intensity resources led to patients overwhelmingly receiving a low-intensity, self-supported internet based treatment, partly because more experienced clinical staff were heavily engaged in assessments to support stratification, and were thus unable to deliver more than a handful of high-intensity treatments.

In marked contrast to site A, site B developed a more stepped model. Few patients were allocated immediately to high-intensity treatment and the overall ratio of low- to high-intensity treatments was 2.6:1, the opposite pattern to site A. Of those patients who were allocated to and subsequently received a treatment, one-third received high-intensity treatment compared to two-thirds in site A. In site B, where entry was not controlled by experienced professionals, these experienced workers were more able to utilise their time in the delivery of high-intensity treatment.

Site D was a complex mixture of 'stepped' and 'stratified' delivery. Although the ratio of low- to high-intensity treatments actually received by patients favoured low-intensity interventions (1.4:1), many more patients were assessed at a high-intensity level. The main differentiating factor in site D was the ability of GPs to make a direct referral to high-intensity primary care psychology. As a consequence, there were two very clear entry points for patients. Although more patients were referred directly to low-intensity compared to high-intensity workers, a considerable number of patients could omit this step.

In summary, the ratio of low- and high-intensity treatments received by patients varied across the sites and ranged from 22:1 (site C including unsupported step one activity), through 2.1:1 (site B), 1.4:1 (site D) to 0.5:1 (mainly high-intensity treatments in site A). In essence, this means that of people treated by site C, 20 times more patients received a low- rather than a high-intensity treatment, whereas in site A twice as many patients received high- as opposed to a low-intensity treatment, a forty-fold allocation difference between the two sites. Furthermore, between sites, the numbers of patients allocated directly to high-intensity treatment varied from 3% to 45%. Rates of stepping up to eventual high-intensity treatment were less than 10% in all sites, although a small number of patients moved from step three to step four within the high-intensity phase of their treatment.

For the three sites where we have referral data, all 'lost' considerable numbers of patients between referral and assessment, ranging from 21% to 34% of referrals, and a similar number were not treated after assessment (Figure 4). Referrals that were not assessed were never seen by the services at all, and we have no data on what happened to these patients. An unscheduled discontinuation rate (*i.e*., where patients drop out of treatment without agreement with a therapist) of 30% was common across treatment steps and sites, although there were variations between sites and steps. As noted in the methods section, the low rates of both scheduled (*i.e*., agreed) and unscheduled discontinuation in high-intensity treatment in some sites are most likely a product of the longer treatment times for high-intensity resulting in a proportion of patients remaining in treatment at the project end date and whose final pathway status was not recorded.

## Discussion

We have reported throughput data on almost 8,000 patients referred to four NHS sites operating stepped care services for common mental health problems, mapping patients' entry and exit from the systems as well as recording the types of therapeutic inputs received. Our data illustrate the considerable variation in the design and implementation of care systems in response to the recommendation for stepped care in the NICE guidelines [8], which lacked explicit detail concerning the optimal model for delivery.

The variation in models was significant. Although it is helpful to place them on an operational continuum from stepped to stratified, this categorisation does not do sufficient justice to their complexity and diversity. While the design of the systems as 'stepped' or 'stratified' is a key dimension that dramatically influences the performance of stepped care systems, staff availability and professional referral behaviour can subvert initial plans in important ways. For example, lack of experienced workers meant that site C became essentially a low-intensity-only service, whereas site D saw a 'stepped' model bypassed by a significant number of patients, although this might also be influenced by patient population characteristics, such as the proportion of patients diagnosed with post traumatic stress disorder where low-intensity treatments are not known to be effective.

In contrast, however, there were some interesting similarities in patient flow between sites. The levels of attrition between referral and assessment have been observed previously in stepped care services [14-16] and are a well-known phenomena in psychological therapies services [17-19]. In the two sites where there was closer balance between low- and high-intensity provision (sites B and D), we observed a consistent 'stepping up' rate of less than 10%, similar to that reported in one of the first Improving Access to Psychological Therapies (IAPT) demonstration sites over three years [16].

Scheduled completion rates for all treatments were variable between sites and steps (Figures 2 and 3). Site D achieved less treatment completion at high-intensity, where only one-third of cases were recorded as completing their high-intensity treatment, although our data are limited by project end dates as cited earlier. Nonetheless, low-intensity treatment is intrinsically shorter than high-intensity treatment, and it is conceivable that patients will be more likely complete a shorter course of low-intensity treatment than the longer high-intensity option. This observation requires further investigation since the concept that treatments should be 'least restrictive' for the patient [5,9,20] is at the heart of the original concept of stepped care.

### Limitations

Our study is of four 'early implementer' sites attempting to reconfigure their services to more closely reflect NICE guidelines [3]. As a consequence, the data may have limited generalisability. Missing demographic data (a product of poor local information systems and haphazard clinical data entry highly prevalent in UK mental health services) limits knowledge of the characteristics of the populations served by these sites.

A more significant limitation is our inability to utilise clinical outcome data to ascertain the outcomes for patients, rather than their flow through the systems. Clinicians and information systems were unable to provide us with this data. Recent evaluations of Australian 'Better Access' mental health systems have similarly suffered from a lack of outcome data, basing published evaluations on a mere 15% of all patients treated [21]. However, it should be noted that the bulk of treatments delivered were evidence-based, as summarised in the NICE guidelines [8], and our analysis assumes that outcomes would be broadly in line with those reported in the guidelines. However, this remains to be confirmed, especially in the context of a stepped care system where patients may receive a number of treatments in a serial fashion.

That clinical services do not use routine data is of greater concern, given the supposed centrality of outcome monitoring in stepped care to support clinical decision-making and ensure that services are responsive when patients do not benefit from initial 'steps.' The assessment of treatment effect to aid clinical decision-making appears deficient in these services. However, the use of formal psychometric measures is not the only way to assess treatment progress. Clinical judgement may be applied by mental health workers using clinical interview assessments. Clearly, for research purposes this is less easy to quantify without routine outcome measures, but their absence does not necessarily invalidate the stepped care process given our patient pathway data. Whether the use of such measures within a formal clinical decision-making algorithm would have reduced the variation in patient pathways could be a potential subject for further research.

We were also ignorant as to any additional service options available to patients and referrers outside the services we studied. Other resources available may alter who is referred to these services and we cannot assume that the proportions of patients 'needing' step two or step three interventions would be the same in all sites.

### Implications

The study highlights variability in the implementation of stepped care for depression. This is to be expected, to the degree that the NICE guidelines were not explicit about a number of issues, and provided no formal 'blueprint' for the organisation and delivery of services. Our observations of variability are confirmed by the report on the first-year IAPT service [22]. Of course, some local variation is desirable, but the very different service delivery models may not be desirable in the long term. It is important that evidence from implementation studies such as that reported here impact on later iterations of the depression guidelines, to maximise standardisation where appropriate and ensure that models of service delivery have a solid basis in evidence, complementing the evidence base relating to the health technologies which are delivered within these services.

Our data suggest that the principal driver of patient flow through stepped care systems is the allocation to initial treatments. The rate of stepping up was low, no matter how the patients were assessed or how many were allocated directly to high-intensity treatment. Not dissimilar proportions of patients were stepped up in systems which allocated large numbers directly to high-intensity treatments, allowed referrers to make direct referral to high-intensity treatment or direct most patients to low-intensity treatment. The two services which stepped fewer patients from low- to high-intensity treatment included one where lack of resources led to very little high-intensity treatment provision, and another where initial allocations to high-intensity treatment was almost 50% of referrals assessed.

Although service planners may seek to design services that reflect their desired balance between stepping and stratification, they must be aware that patient flow is highly sensitive to other factors, including the background of the workers at each step of the service. Triage or assessment by a professionally qualified workforce may lead to more people receiving high-intensity treatment, providing that option is available. Service managers may need to plan on the basis that whatever the initial allocation rates of patients to low- or high-intensity treatments, providing sufficient high-intensity resource is available, less than 10% of patients may be stepped up from low- to high-intensity treatment. It is, therefore, important to resource all available steps sufficiently to allow patients to be stepped up from low- to high-intensity treatment and to prevent situations arising where patients might be inappropriately 'held' at a low-intensity step.

Finally, stepped care systems do not seem to differ from the often observed attrition rates to psychological therapies at all stages in the patient pathway. Access to care has not traditionally received the same research focus as issues of treatment effectiveness. That is now beginning to change [7,23,24] but there is an urgent need to understand the reasons for these levels of attrition and ensure the findings are used to inform the design of stepped care systems in the future.

Although implementation science is relatively new to the evidence-based movement, its use in ensuring that effective treatments and organisational models are put in place consistently is now recommended by research funders [25]. Stepped care would seem to be a prime example of a recommended organisational system idea being interpreted and applied in very different ways. The application of core principles of implementation science (such as targeted planning, implementation strategies and clear activity specification) around service organisation is urgently required.

## Competing interests

The authors declare that they have no competing interests.

## Authors' contributions

DAR, PB, and SG conceived the study and were members of the study investigator committee which designed and oversaw the research methods, along with MU, JC, SP, KL, JL, LO, RP, and DT. CP, AW, SH, AS, and CV managed data collection and analysis. DR and PB drafted the manuscript. All authors read and approved the final manuscript.
